# Mycoplasma pneumoniae infections serve to enhance the risk of subsequently developing severe allergic diseases by chronically abrogating Treg-mediated suppression

**DOI:** 10.1186/2045-7022-4-S3-P109

**Published:** 2014-07-18

**Authors:** Ryo Takahashi, Yukiko Ushigome, Tetsuo Shiohara

**Affiliations:** 1 Kyorin University Graduate School of Medicine, Division of Flow Cytometry, Japan; 2 Kyorin University School of Medicine, Department of Dermatology, Japan

## Background

Although the relation of Stevens-Johnson syndrome (SJS) and Mycoplasma pneumoniae (MP) infection has been well known, it remains unknown how MP infection contributes to SJS development.

## Method

We investigated the frequencies, phenotype and function of Tregs in the peripheral blood of MP infection and viral infections (measles virus, varicella-zoster virus, parvovirus B19, and EBV) patients at onset and after clinical resolution, and healthy controls. The cytokine production from CD4+ T cells stimulated with PMA/ionomycin and Toll-like 2 and 4 specific cytokine productions from monocytes was measured by FACS. Treg suppressive function was measured by 3H-thymidine incorporation.

## Results

During the acute stage of MP and viral infections, the functional activity of Tregs to suppress T cell proliferation was profoundly impaired, although frequencies of CD25++Foxp3+ Tregs in CD4+ T cells from patients of MP, varicella-zoster virus, and parvovirus B19 infections were not significantly altered. Upon resolution, however, the impaired functional activity of Tregs was restored in viral infections. In contrast, abrogation of Treg function lasted 3 months to a year after MP infection.

Next, we investigated phenotype of Tregs in patients of MP infection. A recent study has indicated that Foxp3+CD4+ T cells can be classified into functionally distinct subpopulations based on CD45RA and Foxp3 expression: CD45RA+Foxp3+ natural Tregs, CD45RAFoxp3++ induced Tregs, and CD45RAFoxp3+ nonsuppressive T cells. Although expression level of CTLA-4, CD127, and CD39 on Foxp3+CD4+ T cells did not change compared with controls, the percentage of CD45RAFoxp3+ in Foxp3+CD4+ T cells was significantly increased in the resolution stage of MP infection. The percentage of Th1 and Th2 but not Th17 was significantly increased during the resolution stage of MP infection.

Our recent study has reported that monocytes can control peripheral Treg development. Both of IL-6 and IL-10 production from CD14+ monocytes upon TLR-2 but not TLR-4 stimulation was significantly increased during the resolution stage of MP infection. These results suggest that the unique cytokine profile of monocytes upon TLR2 stimulation might be associated with induction CD45RAFoxp3+ T cells.

## Conclusion

Our findings suggest that MP infections could serve to enhance the risk of subsequently developing severe allergic diseases by chronically abrogating Treg-mediated suppression.

